# A Novel Synthetic Odorant Blend for Trapping of Malaria and Other African Mosquito Species

**DOI:** 10.1007/s10886-012-0088-8

**Published:** 2012-03-18

**Authors:** Wolfgang R. Mukabana, Collins K. Mweresa, Bruno Otieno, Philemon Omusula, Renate C. Smallegange, Joop J. A. van Loon, Willem Takken

**Affiliations:** 1 International Centre of Insect Physiology and Ecology, PO Box 30772, GPO Nairobi, Kenya; 2 School of Biological Sciences, University of Nairobi, PO Box 30197, GPO Nairobi, Kenya; 3 Laboratory of Entomology, Wageningen University and Research Centre, PO Box 8031, 6700 EH Wageningen, The Netherlands; 4 Present Address: Enza Zaden R&D B.V., Enkhuizen, The Netherlands

**Keywords:** Odor-guided behavior, Synthetic odor blend, Mosquito, *Anopheles gambiae*, Sampling, Isovaleric acid, 4,5-dimethylthiazole, 2-methyl-1-butanol, 3-methyl-1-butanol

## Abstract

Estimating the biting fraction of mosquitoes is of critical importance for risk assessment of malaria transmission. Here, we present a novel odor-based tool that has been rigorously assessed in semi-field assays and traditional African villages for estimating the number of mosquitoes that enter houses in search of a blood meal. A standard synthetic blend (SB) consisting of ammonia, (S)-lactic acid, tetradecanoic acid, and carbon dioxide was complemented with isovaleric acid, 4,5 dimethylthiazole, 2-methyl-1-butanol, and 3-methyl-1-butanol in various combinations and concentrations, and tested for attractiveness to the malaria mosquito *Anopheles gambiae*. Compounds were released through low density polyethylene (LDPE) material or from nylon strips (nylon). Studies were done in a semi-field facility and two traditional villages in western Kenya. The alcohol 3-methyl-1-butanol significantly increased the attraction of SB. The other compounds proved less effective or inhibitory. Tested in a village, 3-methyl-1-butanol, released from LDPE, increased the attraction of SB. Further studies showed a significantly enhanced attraction of adding 3-methyl-1-butanol to SB compared to previously-published attractive blends both under semi-field and village conditions. Other mosquito species with relevance for public health were collected with this blend in significantly higher numbers as well. These results demonstrate the advent of a novel, reliable odor-based sampling tool for the collection of malaria and other mosquitoes. The advantage of this odor-based tool over existing mosquito sampling tools is its reproducibility, objectiveness, and relatively low cost compared to current standards of CDC light traps or the human landing catch.

## Introduction

Blood-questing mosquitoes mainly rely on olfactory cues to locate their hosts (Takken, 1991; Costantini et al., 1996; Takken and Knols, 1999). For the principal African malaria vectors, *Anopheles gambiae* Giles *sensu stricto*, *An. arabiensis* Patton and *An. funestus* Giles, these cues are strongly reminiscent of those released by humans i.e., the principal source of the mosquitoes’ blood meals (Gillies and Coetzee, 1987). Odorants from human skin and carbon dioxide from breath are particularly important (Costantini et al., 1996; Mukabana et al., 2004; Spitzen et al., 2008). The anthropophilic malaria mosquito *Anopheles gambiae sensu stricto* (hereafter termed *An. gambiae*) primarily takes blood meals on humans, whereas its close sibling *An. arabiensis* is more opportunistic, feeding on humans and animals. This difference in host preference is expressed clearly in odor-guided behavior, where *An. arabiensis* responds significantly stronger to carbon dioxide from breath than does *An. gambiae* (Costantini et al., 1996). These behavioral differences also are reflected in the role of these species as malaria vectors, *An. gambiae* being the principal species even though *An. arabiensis* is equally susceptible to the *Plasmodium* parasite but, due to its different feeding behavior, is of less importance as a vector. Host preference, and hence the selection of hosts based on their odorants, is of principal importance to understand the role of these mosquitoes in malaria epidemiology.

Given the strong association of *An. gambiae* with humans, unraveling the odor cues that mediate this behavior is of scientific as well as practical importance. For *An. gambiae* the principal olfactory cues of humans originate from the feet (De Jong and Knols, 1995), and recent work has demonstrated that these cues are partially produced by the microbial flora present on the feet (Verhulst et al., 2010). From these studies, several chemical compounds have been identified that play a critical role in the odor-mediated behavior of *An. gambiae* (Smallegange et al., 2005, 2009; Verhulst et al., 2011a). Detailed information on the role of these compounds allows for the development of synthetic blends that can be used to better understand the host-seeking behavior of this mosquito. Such blends also have the potential to be used for mosquito surveillance or intervention through mass trapping.

Studies on the development of kairomones for malaria vectors have demonstrated strong behavioral responses to synthetic blends of human odorant compounds (Smallegange et al., 2010a). In laboratory and semi-field studies, these blends attract a large proportion of host-seeking mosquitoes, but when tested against a natural human host, or against natural human odorants released from a nylon matrix, these blends demonstrate poor competitive characters compared to the natural odorants. This suggests that either the concentration of the odorants in the blend was insufficient or that one or more compounds to make the blend sufficiently competitive were missing (Smallegange et al., 2005, 2010a; Verhulst et al., 2011a). Recent progress, however, has demonstrated the development of odor blends that approach the attractiveness of natural human skin odorants (Okumu et al., 2010b).

In addition, there is sometimes a mismatch between laboratory behavioral results and field trials, which can be caused by differences in concentration or spatial effects (Verhulst et al., 2009, 2011a). Assessment of existing synthetic attractant compounds under semi-field and field conditions provides a potential for the development of technologies that can be used for sampling and control of malaria mosquitoes (Kline, 2006; Jawara et al., 2009; Okumu et al., 2010b). The current study was designed to evaluate the attractiveness of selected synthetic blends and human hosts to host-seeking mosquitoes in western Kenya, with emphasis on the malaria vectors *An. gambiae sensustricto* and *An. arabiensis*. Thus, the comparative trapping efficacy of the attractant blends and the physiological status of the mosquitoes trapped were investigated.

## Methods and Materials

### Mosquitoes

Behavioral responses of mosquitoes to synthetic attractants were evaluated under field and semi-field conditions. The semi-field experiments utilized a laboratory colony of the Mbita strain of *An. gambiae*. Aquatic stages of the mosquitoes were reared under ambient atmospheric conditions in screen-walled greenhouses at the Thomas Risley Odhiambo (TRO) campus of the International Centre of Insect Physiology and Ecology (*icipe*) located near Mbita Point township in western Kenya. Mosquito eggs were placed in plastic trays containing filtered water from Lake Victoria. All larval instars were fed on Tetramin® baby fish food supplied three times a day. Pupae were collected daily, transferred to adult holding rooms, and placed in mesh-covered cages (30 × 30 × 30 cm) prior to adult emergence. Adult mosquitoes were fed on 6% glucose solution through wicks made from adsorbent tissue paper. Mosquitoes used for semi-field experiments were placed in mosquito-gauze covered plastic cups and starved for 8 hr. They had no prior access to a blood meal. Only water, availed on wet cotton towels placed on top of mosquito holding cups, was provided during starvation. All semi-field experiments were carried out at night (2030–0630 h) inside the screenhouses (Verhulst et al., 2011b). Two hundred, 8-h-starved mosquitoes were used each experimental night.

### Compounds Used to Constitute Odorant Blends

All chemicals used to constitute the synthetic attractant blends in this study, with the exception of carbon dioxide, water, sugar, and yeast, were purchased from Sigma-Aldrich Chemie GmbH (Germany). The chemicals included propionic acid (99.6%), butanoic acid (≥99.9%), pentanoic acid (>99%), heptanoic acid (98%), octanoic acid (≥99%), tetradecanoic acid (≥99%), ammonia solution (purity 25%), (S)-lactic acid (85%), isovaleric acid (99.8%), 4,5-dimethylthiazole (97%), 2-methyl-1-butanol (99%; a racemic mixture of the R and S isomers of unknown ratio), and 3-methyl-1-butanol (purity ≥ 98.5%). Carbon dioxide was produced by mixing 250 g sucrose (Sony sugar company Limited, Kenya), 17.5 g dry yeast (Angel Yeast company limited, China), and water (2 L) as described in Smallegange et al. (2010b).

### Study Sites

Field studies were carried out in Lwanda and Kigoche villages of Homa Bay and Kisumu counties of western Kenya, respectively. Lwanda village is located on the southern shore of the Winam Gulf of Lake Victoria (00°28′28″S, 34°17′22″E) at an altitude of 1,169 m above sea level (Verhulst et al., 2011a). Average rainfall and relative humidity are 1,200 mm and 65%, respectively. The mean temperatures vary between 18°C and 34°C. Hoof prints of cattle and night-grazing hippopotami provide excellent mosquito breeding sites in Lwanda. Fishing and livestock keeping are the main occupation of the local inhabitants. Kigoche village lies adjacent to the Ahero rice irrigation scheme (00°08′19″S, 34°55′50″E) at an altitude of 1,160 m above sea level. Kigoche has an average annual rainfall of 1,000–1,800 mm and an average relative humidity of 65%. Mean annual temperatures in the area vary between 17°C and 32°C. Rice cultivation is the main occupation of the inhabitants. Most houses in the two villages are mud-walled with open eaves, have corrugated iron-sheet roofs, have no ceiling, and are either single- or double-roomed. Eaves, about one foot wide, increase ventilation in the houses and form the predominant entry points for mosquitoes (Snow, 1987; Lindsay and Snow, 1988). Malaria caused by *Plasmodium falciparum* is endemic in the two villages. The villages experience two rainy seasons: between April–June and September–October. During these periods, mosquito breeding grounds proliferate, and mosquito populations rapidly increase in size. Cattle, goats, chicken, dogs, cats, and a few sheep constitute the domestic animal population, with cattle being most abundant. Maize, millet and sorghum are cultivated at subsistence level in Lwanda, whereas rice is a main cash crop in Kigoche.

In both villages, trapped mosquitoes were morphologically identified using the keys published by Gillies and Coetzee (1987), counted, and the data entered in MS Excel spreadsheets. Culicine mosquitoes were identified up to genus level, and anophelines into *An. gambiae* sensu lato, *An. funestus* and other anopheline species. Abdominal statuses of female mosquitoes were categorized as unfed, blood-fed, or gravid. Mosquitoes belonging to the *An. gambiae* complex were identified to species using a ribosomal DNA Polymerase Chain Reaction assay (Scott et al., 1993).

### Constituting Prototype Blends and Evaluating Their Attractiveness to *Anopheles gambiae*

Prototype blends were made by adding components to a standard attractive blend (standard blend, SB) consisting of ammonia, (S)-lactic acid, and tetradecanoic acid (Smallegange et al., 2005, 2009). The standard blend was augmented with locally-made carbon dioxide plus individual additional candidate compounds or groups of 2–4 of these compounds. The additional compounds included isovaleric acid, 4,5-dimethylthiazole, 2-methyl-1-butanol, and 3-methyl-1-butanol. These compounds were selected following two studies in which a total of 39 chemical compounds were evaluated for their attractiveness to *An. gambiae* (Verhulst et al., 2011b; Smallegange et al., 2012). All compounds except carbon dioxide were delivered to experimental mosquitoes by using low density polyethylene sachets (LDPE). To prepare a sachet, a tube foil of LDPE material was cut (35 × 30 mm), thermally sealed at one end before pipetting 1,000 μl of a pure compound through the open end of the sachet. The sachet was subsequently sealed, confining the candidate compounds within an area of 25 × 25 mm. Only a single candidate compound was placed in an individual sachet. The wall thickness of the LDPE material used was 0.1 mm (isovaleric acid and 3-methyl-1-butanol), 0.2 mm (2-methyl-1-butanol), 0.03 mm (ammonia solution, 4,5 dimethylthiazole, and tetradecanoic acid), and 0.05 mm ((S)-lactic acid). Several sachets, varying in number according to the number of chemical compounds constituting a specific prototype blend, were placed onto a hook (Verhulst et al., 2009), which was subsequently inserted inside the outlet tube of a MM-X trap (American Biophysics Cooperation, RI, USA). Dual-choice tests comparing mosquito behavioral responses to a total of 15 prototype blends, with different combinations of the four candidate compounds + SB vs. SB alone then were conducted in the semi-field screenhouse facility by placing MM-X traps in diagonal corners (Verhulst et al., 2011a). The experiments were replicated four times.

### Attraction of Wild Mosquitoes to the Best Prototype Blend against Existing Alternatives

The prototype blend attracting the highest numbers of *An. gambiae* mosquitoes in the screenhouse was evaluated in Lwanda village and compared with the attractiveness of other blends, which we had tested in previous field experiments (Okumu et al., 2010b). The number of mosquitoes attracted to each one of three blends including Ifakara blend 1 (IB1; Okumu et al., 2010a), standard blend (Smallegange et al., 2005, 2009) and a best, newly-formulated prototype blend namely Mbita blend (MB) was recorded. The blends were dispensed via LDPE sachets or nylon strips. The nylon fabric was made of 15 denier microfibers. The textile composition was 90% polyamide and 10% spandex (Bata Shoe Company, Kenya). The nylon strips were cut into narrow pieces each measuring 26.5 cm long and 1.0 cm wide. Each strip then was soaked in separate solutions of the attractants until saturation. Treatments included blend IB1 dispensed via nylon strips, IB1 dispensed via LDPE sachets, SB dispensed via nylon strips, SB dispensed via LDPE sachets, and MB dispensed via LDPE sachets. Blend IB1 consisted of propionic acid (0.01%; LDPE thickness 0.2 mm), butanoic acid (1%; LDPE 0.2 mm), pentanoic acid (0.0001%; LDPE 0.2 mm), 3-methyl butanoic acid (0.000001%; LDPE 0.2 mm), heptanoic acid (0.0001%; LDPE 0.1 mm), octanoic acid (0.0001%; LDPE 0.1 mm), tetradecanoic acid (0.00025%; LDPE 0.03 mm), ammonia (2.5%; LDPE 0.03 mm), (S)lactic acid (85%; LDPE 0.05 mm), distilled water (LDPE 0.2 mm), and carbon dioxide (∼63.23 ± 2.82 ml/min). An unbaited MM-X trap acted as the negative control. All blends were dispensed from MM-X traps hung outside the bedroom window (for details: see Verhulst et al. (2011b)). A total of eight village houses were selected and experiments carried out from 1830 to 0630 h each night for 40 nights. Treatments were rotated around the eight houses to eliminate positional effects. The selected houses were mud-walled, had open eaves and corrugated iron sheet roofs. The houses, occupied by owners (one person per house) throughout the night, were located at least 25 m. apart (Hill et al., 2007).

### Optimizing the Most Attractive Prototype Blend on Nylon Strips by Varying the Concentration of 3-Methyl-1-butanol

The blend that attracted most mosquitoes compared to the standard blend was selected and adapted for release on nylon strips, which are known to yield higher mosquito catches than LDPE sachets (Okumu et al., 2010a). The blend, named Mbita blend or MB consisted of 3-methyl-1-butanol, tetradecanoic acid, ammonia solution, (S)-lactic acid, and carbon dioxide. The most effective dilutions for tetradecanoic acid (0.00025%), ammonia (2.5%), (S)-lactic acid (85%) had been determined previously (Okumu et al., 2010b). Carbon dioxide was released at ∼63.23 ± 2.82 ml/min. Thus, the optimal dilution for dispensing 3-methyl-1-butanol on nylon strips was determined experimentally under semi-field conditions using MM-X traps. Binary assays evaluating mosquito behavioral responses to SB with all constituents availed at their optimally attractive concentrations vs. SB plus 3-methyl-1-butanol offered at variable dilutions (100%, 10%, 1%, 0.01% and 0.0001% and 0.000001% *v*/*v*) were run.

### Efficacy of Attracting Mosquitoes to Optimized Blend Under Semi-field Conditions

The efficacy of attracting host-seeking mosquitoes using MB was evaluated by testing it against Ifakara Blend 1 (IB1), the standard blend (SB), and a control (unbaited trap) under semi-field conditions. A fully replicated 4 × 4 Latin Square experiment was used. All blends were dispensed on nylon strips by pipetting 1,000 μl of each component of a blend on a separate strip (measuring 26.5 × 1.0 cm). The 3-methyl-1-butanol component of MB was used at a dilution of 0.000001%.

### Capacity of Trapping Wild Mosquitoes Using Optimized Blend Vs. Existing Alternatives

In Kigoche village, five houses spaced apart at a distance of at least 25 m and at least 100 m away from rice paddies were selected for the study. A 5 × 5 Latin square experimental design preceded by a 5 d trial period was run to assess the potential of MB in attracting malaria and other mosquito vectors. The treatments, assigned to each of the five houses on rotational basis per night, included a human host, IB1, SB, MB, and an empty house i.e., the control. All components of the synthetic attractants, with the exception of carbon dioxide, were delivered via nylon strips (Okumu et al., 2010a) and dispensed using MM-X traps. The traps were suspended 15 cm above a bed inside unimpregnated mosquito nets, and were operated using 12 volt batteries. One of the fans on the MM-X trap was disabled to prevent it from trapping mosquitoes. Mosquitoes were trapped by hanging an unlit CDC miniature light trap (Model 512; John W. Hock Company, Gainesville Fl., USA) operated on 6 V batteries (Gaston Battery Industry Ltd, China) beside and at 15 cm (Jawara et al., 2009) above the odor outlet tube of the MM-X trap, outside the bed net. Both MM-X and CDC light traps were hung on the foot end region of the beds in all cases (Mboera et al., 2000). Only one MM-X trap and one CDC light trap were used in houses where mosquito catches were based on synthetic attractants. Only one CDC light trap was used in houses occupied by human subjects. The volunteer slept inside a well tucked-in bed net with a CDC light trap suspended besides it. Whereas a total of five adult men aged 18–25 years volunteered to participate in the study, only one individual participated per night. The volunteers rotated over the treatments according to a random design. Each experimental night lasted from 1900 to 0630 h. After the experiments traps were disconnected from the batteries, the trapped mosquitoes were taken to the laboratory, where they were killed by freezing at −4°C. Traps were cleaned with 30% methanol solution before being reused. During experimental nights, the residents of the study houses were not present. Other houses in the village were occupied normally.

### Ethical Considerations

Consent for houses to be used in the study was obtained from the household heads and the local administration prior to the start of the study.

### Statistical Analysis

Dispersion tests, performed to establish whether means equaled variances (Grafen and Rosie, 2005), were employed to determine if the count data indeed assumed a Poisson distribution. Henceforth, the number of mosquitoes attracted to the different sources of behavioral stimuli (human subjects, control, or the synthetic attractants) was modeled as a proportion of the total number of mosquitoes recovered from the different treatments. All statistical analyses were carried out using Generalized Linear Models (Agresti, 1990) in which data were transformed to assume linearity using a logarithmic link function. This approach allowed for differences in attractiveness between treatments to be determined. All fitted models were followed by *post hoc t*-tests, to assess levels of statistical difference between pairs of competing blends assessed through the binary assays. It was assumed that mosquitoes already trapped did not affect subsequent mosquitoes entering. All data were analyzed using the General statistical software program (GenStat Discovery Edition 3) (Payne, 1986).

## Results

### Constituting Prototype Blends and Evaluating Their Attractiveness to *Anopheles gambiae*

Adding 3-methyl-1-butanol to the standard blend of ammonia, (S)-lactic acid, tetradecanoic acid, and carbon dioxide either singly or in combination with 2-methyl-1-butanol formed the two most potent attractant blends for host-seeking *An. gambiae* mosquitoes (catching 72% of those released) relative to the standard blend on its own under semi-field conditions (Table 1). Neither adding 2-methyl-1-butanol to the basic blend, nor adding it to 3-methyl-1-butanol enhanced attractiveness. The blend containing 3-methyl-1-butanol plus components of the standard blend was considered the most potent synthetic attractant for *An. gambiae*. The prototype product, termed Mbita blend (MB), consisted of 3-methyl-1-butanol (released in 0.1 mm-LDPE sachets), tetradecanoic acid (0.03 mm-LDPE), ammonia solution (0.03 mm-LDPE), (S)-lactic acid (0.05 mm-LDPE), and carbon dioxide (∼130 ml/min). As adding isovaleric acid and 4,5 dimethyl-thiazole to the standard blend diminished the numbers of *An. gambiae* attracted (Table 1), these compounds were excluded from the prototype blend.

**Table 1:** Mean (±SE) mosquito catches per night and levels of statistical difference (*P*-value) between 15 prototype synthetic blends vs. a standard blend (SB) of ammonia, (S)-lactic acid, tetradecanoic acid, and carbon dioxide. Each of the compounds except carbon dioxide was dispensed from a LDPE-sachet in pure form. *N* is the number of replicates (nights) and *n* the total number of mosquitoes trapped out of a total of 800 released.% response equals n/800. Compound 1, 2, 3, and 4 are isovaleric acid, 4,5 dimethyl-thiazole, 2-methyl-1-butanol, and 3-methyl-1-butanol, respectively

Description of synthetic blend	*N*	*n*	% response	Mosquito trap catches (mean ± SE)		*P*-value
				Standard blend (SB)	Synthetic blend	
1. SB + compound 1	4	346	43	62.20 ± 3.70	24.25 ± 4.25	0.001
2. SB + compound 2	4	338	42	46.75 ± 8.83	37.75 ± 6.33	0.05
3. SB + compound 3	4	334	42	38.67 ± 20.42	43.00 ± 12.97	0.037
4. SB + compound 4	4	577	72	59.00 ± 7.00	85.20 ± 9.8	0.001
5. SB + compound 2&3	4	508	64	67.67 ± 5.78	66.50 ± 11.2	0.240
6. SB + compound 1&2	4	482	60	70.67 ± 8.35	57.25 ± 4.40	0.025
7. SB + compound 1&3	4	444	56	68.33 ± 12.14	44.75 ± 9.38	0.001
8. SB + compound 1&2&3	4	462	58	49.33 ± 17.53	61.20 ± 12.7	0.008
9. SB + compound 3&4	4	579	72	57.00 ± 6.51	85.00 ± 11.2	0.001
10. SB + compound 1&4	4	450	56	71.00 ± 12.9	31.75 ± 9.72	0.001
11. SB + compound 2&4	4	390	47	45.00 ± 7.21	46.25 ± 9.04	0.748
12. SB + compound 1&2&4	4	253	37	52.00 ± 13.32	11.75 ± 2.87	0.001
13. SB + compound 1&3&4	4	391	49	52.67 ± 4.81	54.00 ± 7.63	0.645
14. SB + compound 2&3&4	4	481	60	63.33 ± 14.77	56.25 ± 16.47	0.388
15. SB + compound 1&2&3&4	4	219	27	28.00 ± 9.81	25.75 ± 13.49	0.350

### Attraction of Wild Mosquitoes to the Best Prototype Blend Tested Against Existing Alternatives

A total of 1,286 mosquitoes were trapped in Lwanda village over a period of 40 d from 28 April 2010 to 11 June 2010. The mosquitoes included *An. gambiae* s.l. (*n* = 259), *An. funestus* (*n* = 207), *Culex* species (*n* = 544), *Mansonia* species (*n* = 112), *Aedes* species (*n* = 43), and other mosquito species (*n* = 121) (Table 2). Although all synthetic blends caught significantly more *An. gambiae* s.l. than the unbaited traps, no blend attracted significantly more mosquitoes of this species than the other (Table 2). However, the standard blend dispensed from nylon strips attracted comparatively higher numbers of *Mansonia* spp, *Aedes* spp. and *An. funestus* s.l. than the other blends. Ifakara blend 1 (IB1) dispensed using nylon strips attracted comparatively higher numbers of *Culex* spp. and *An. gambiae* s.l. than all the other blends. As this study was performed before the optimal dilution for releasing MB on nylon strips was determined, it was not possible to fully evaluate its competitiveness against other attractants in Lwanda village. Out of the 107 specimens of *An. gambiae* s.l. that were subjected to PCR analysis, 100 could be identified to species level. Ninety six of these were *An. arabiensis*, whereas four were *An. gambiae s.s*..

**Table 2:** Mean numbers of *Anopheles gambiae* s.l., *An. funestus*, *Culex* spp., *Mansonia* spp., *Aedes* spp. and other mosquito species collected indoors per night in Lwanda village, western Kenya. Mosquitoes were attracted to various synthetic attractants dispensed from nylon strips or LDPE sachets mounted in MM-X traps placed under a bed net. *N* = number of replicates (nights); *n* is total number of mosquitoes per taxon caught over 40 nights. Numbers followed by different letter superscripts in the same column differ significantly (*P* < 0.05)

Treatment (delivery)	*N*	*An. gambiae *s.l.	*An. funestus*	*Culex* spp.	*Mansonia* spp.	*Aedes* spp.	Other spp.
Empty (control)	40	0.65^a^	0.55^a^	2.28^a^	0.25^a^	0.10^a^	0.43^a^
IB1 (LDPE)	40	1.30^b^	0.53^a^	1.85^a^	0.53^b^	0.15^ab^	0.43^a^
IB1 (nylon strips)	40	1.43^b^	1.15^b^	2.98^b^	0.55^b^	0.13^a^	0.58^b^
SB (LDPE)	40	1.05^b^	0.58^a^	2.48^a^	0.33^ab^	0.25^a^	0.38^a^
SB (nylon strips)	40	1.03^b^	1.73^c^	1.88^a^	0.60^b^	0.38^b^	0.40^a^
MB (LDPE)	40	1.03^b^	0.58^a^	2.15^a^	0.55^b^	0.08^a^	0.83^c^
Total mosquito catches (*n*)		259	207	544	112	43	121

### Optimizing the Most Attractive Prototype Blend on Nylon Strips by Varying the Concentration of 3-Methyl-1-butanol

The overall combined response of the mosquitoes to the blends ranged from 39% to 68% (Table 3). Dilutions of 3-methyl-1-bunanol from pure compound to 10,000 times were significantly (*P* < 0.001) in favor of the standard blend, except for the 1,000-fold dilution, for which no difference in attractiveness between the two blends was found. Only with the highest dilution (100,000) did significantly more (*P* < 0.05) mosquitoes respond to the augmented blend than to the standard blend. This blend (MB) subsequently was chosen for further evaluation.

**Table 3:** Mean number ± SE of *Anopheles gambiae* caught per night in MM-X traps baited with a synthetic blend containing various dilutions of 3-methyl-1-butanol plus a standard blend vs. the standard blend alone, dispensed from nylon strips, under semi-field conditions. *P*-values give the significance of statistical difference between the catches. *N* is the number of replicates (nights) and *n* the number of mosquitoes trapped

Dilution (%)	*N*	*n*	% response	Mosquito trap catches (mean ± SE)		*P*-value
				SB	Synthetic blend	
Pure compound (99.9)	4	547	68	77.50 ± 16.97	59.25 ± 2.14	0.002
10.0	4	340	43	52.00 ± 18.48	33.00 ± 11.20	0.001
1.0	4	474	59	68.25 ± 13.12	50.25 ± 17.80	0.001
0.1	4	412	52	53.25 ± 4.290	49.75 ±14.42	0.490
0.01	4	307	39	45.25 ± 6.24	31.50 ± 4.05	0.002
0.001	4	379	47	42.25 ± 12.59	52.25 ± 8.09	0.040

### Efficacy of Attracting Mosquitoes to Optimized Blend Under Semi-field Conditions

The attraction efficacy of MB vs. IB1, SB, and a control (unbaited trap) was evaluated over a period of 16 nights i.e., from 30 November 2010 to 16 December 2010. Out of the total of 3,200 mosquitoes used for these experiments, 2,152 were captured in response to the various treatments. There was a significant effect (GLM; *P* < 0.001) of treatment on mosquito trap catches. Whereas all the synthetic blends attracted significantly more mosquitoes than the unbaited control (*P* = 0.001), MB attracted more mosquitoes than blends IB1 (*P* = 0.001) and SB (*P* = 0.001). Blend IB1 attracted more mosquitoes than SB (*P* = 0.001). Results of these experiments are shown in Table 4.

**Table 4:** Mean number ± SE of *Anopheles gambiae* caught per night in MM-X traps baited with different synthetic blends under semi-field conditions. Synthetic odors were dispensed from nylon strips placed in the MM-X trap. Numbers with different letter superscripts in the same column differ significantly (GLM; *P* < 0.001). *N* is the number of replicates (nights) and *n* the total number of mosquitoes trapped. The effect of treatment (*P* values) on overall mosquito responses is also shown

Blend (delivery)	*N*	*n*	Mosquito trap catches (mean ± SE)	Treatment (*P*-value)
Control (no odors)	16	58	3.62 ± 0.81^a^	0.001
IB1	16	691	43.2 ± 4.4^b^	0.001
SB	16	525	32.8 ± 5.4^c^	0.001
MB	16	878	54.9 ± 8.1^d^	0.001

### Capacity of Trapping Wild Mosquitoes Using Optimized Blend Vs. Existing Alternatives

The competitiveness of MB over other baits in attracting malaria and other wild mosquitoes was evaluated in Kigoche village for 30 d spanning the period 01–30 April, 2011. A total of 2,024 mosquitoes including 1,105 (54.6%) *An. gambiae* s.l., 433 (21.4%) *An. funestus*, 201 (9.9%) other *Anopheles* species and 283 (14%) *Culex* species were collected. The collections also included one *Aedes* and one *Mansonia* mosquito representing 0.1% of the collection. The *An. gambiae* s.l. collected included 34 male and 1,071 female (92.8% unfed; 5.3% blood fed, and 1.9% gravid) mosquitoes. It is worth noting that MB collected higher fractions of blood fed *An. gambiae* s.l. (38.6% i.e., 22/57) and *An. funestus* (75% i.e., 6/8) mosquitoes than IB1 and SB. Polymerase chain reaction analysis revealed that the *An. gambiae* complex mosquitoes were 96.67% *An. arabiensis* and 3.33% *An. gambiae s.s*. The *An. funestus* species of mosquitoes included 56 male and 377 female (96.8% unfed; 2.1% blood fed, and 1.1% gravid) mosquitoes. All *Culex*, *Aedes* and *Mansonia* species collected were female. The *Culex* sub-sample consisted of 196 (97.5%) unfed and 5 (2.5%) blood fed mosquitoes. The other *Anopheles* species were neither classified by sex nor abdominal status.

Analysis of the data revealed that houses baited with MB caught 31.5% (*n* = 637) of the mosquitoes (males and females) while those with a human being, IB1, SB, or no host cue collected 26.9% (*n* = 545), 19.6% (*n* = 397), 17.4% (*n* = 352), and 4.6% (*n* = 93) of the mosquitoes, respectively. The catches of *An. gambiae* s.l. (*P* < 0.042), *An. funestus* (*P* < 0.014) and *Culex* species (*P* < 0.041) were significantly influenced by treatment (Table 5). The mean number of female mosquitoes with the exception of *Culex* species collected from the five traditional houses did not differ significantly. The numbers of mosquitoes of all species collected from the empty house were significantly lower than those collected from houses baited with MB (*P* = 0.001), a human being, IB1 (*P* = 0.001), or SB (*P* = 0.001). Mbita blend attracted significantly higher numbers of *An. funestus* and *Culex* species than a human being (*P* = 0.002 and 0.001, respectively) or SB (*P* = 0.001 and 0.005, respectively). However, although MB attracted higher numbers of *An. gambiae* s.l. mosquitoes than IB1 (*P* = 0.001) and SB (*P* = 0.001), these numbers did not differ significantly from those attracted by a human being (*P* = 0.121). Furthermore, although MB attracted more *An. funestus* mosquitoes then IB1 (*P* = 0.001), the number of *Culex* mosquitoes attracted to MB were not different from those attracted to blend IB1 (*P* = 0.140)

**Table 5:** Total pooled number of female mosquitoes and mean number ± SE per species per night trapped in response to different synthetic attractant blends (dispensed form nylon strips) and a human host in Kigoche village, western Kenya. Numbers with different letter superscripts in the same column differ significantly. The number of replicates (*N*) is shown

Treatment	*N*	Total no. of female mosquitoes collected	Mean (±SE) of female mosquito catches per night					
			*An. gambiae* s.l.	*An. funestus*	*Culex* spp.	*Aedes* spp.	*Mansonia* spp.	Other anophelines
Control	30	81	1.2 ± 0.41^a^	0.8 ± 0.28^a^	0.33 ± 0.15^a^	0	0	0.37 ± 0.021^a^
Human	30	527	9.9 ± 2.1^b^	2.93 ± 0.65^b^	1.17 ± 0.28^b^	0	0	3.57 ± 0.96^b^
IB1	30	375	7.13 ± 2.90^c^	2.0 ± 0.39^b^	1.7 ± 0.39^c^	0.03 ± 0.03	0.03 ± 0.03	1.60 ± 0.49 ^c^
SB	30	333	6.27 ± 1.40^c^	2.03 ± 0.59 ^b^	1.27 ± 0.33^b^	0	0	1.53 ± 0.41^c^
MB	30	618	11.2 ± 2.1^b^	4.8 ± 1.26^c^	2.23 ± 0.72^c^	0	0	2.37 ± 0.80^bc^

## Discussion

The mosquito catches in the screenhouse and the village houses consistently showed a higher number of *An. gambiae* when traps were baited with the Mbita blend compared to the Ifakara blend 1 and the standard blend. In Kigoche village, significantly more *An. gambiae* s.l. and *An. funestus*, the main malaria vectors in western Kenya, were caught with MB than with the other two blends, and catches were similar to (*An. gambiae* s.l.) or significantly more than those (*An. funestus*) attracted to a human host. In a previous study in Tanzania, that used experimental huts, Okumu et al. (2010b) reported significantly higher catches of *An. gambiae* s.l. (mostly *An. arabiensis*) with IB1 than with a human host. From both studies, we conclude, therefore, that synthetic blends can be effectively used for sampling African malaria mosquitoes.

The odor-baited technology has a distinct advantage over the widely-used CDC trap + human-under-a-bed-net method introduced by Garrett-Jones and Magayuka (1975), which has since been used as the standard method for assessing entomological inoculation rates and other relevant epidemiological parameters for malaria (Smith et al., 2006; Bousema et al., 2010). The CDC trap was evaluated by Lines et al. (1991) and found to sample approximately two-thirds of the mosquitoes attracted to a human host. Later assessments of the CDC trap reported a similar efficiency but with local variations (Costantini et al., 1998). Given the natural variation in attractiveness of humans to mosquitoes (Smith, 1956; Brouwer, 1960; Knols et al., 1995; Qiu et al., 2006), caused by the variation in human skin odorants and breath (Mukabana et al., 2004; Verhulst et al., 2012), as well as the need for a human host to be present during the all-night collections, the CDC trap plus bed net combination for mosquito trapping has obvious disadvantages. These disadvantages can be overcome by replacing the human host with synthetic bait (over a time frame of at least 1 week), which produces a consistent and constant blend of odorants and hence avoids the variance caused by the human-dependent method of mosquito sampling. Natural variance in human odorants is likely to affect trap catches, and may potentially lead to stronger attractiveness of the natural host than the synthetic blend, but also to considerably lower attractiveness. Future studies should provide more details on these phenomena. Odor-baited traps also can be used in large numbers simultaneously across a study area, which avoids bias caused by spatial effects.

In this study, we examined the use of LDPE as a material for the slow release of odorants, analogous to the successful use of LDPE for attractants of tsetse flies (Green, 1994; Torr et al., 1995). Odorants attractive to tsetse flies and released through LDPE remain active for many months and have been used widely for the mass trapping of tsetse flies in remote areas. Although odorants released through LDPE used in our study proved attractive to anopheline and other mosquitoes, we found that releasing the odorants from nylon strips caused a significantly higher attraction of several of the mosquito species. Okumu et al. (2010a) also reported this effect. It seems likely that the size of the LDPE sachets we employed was too small to provide a sufficiently large surface to release the quantity of odorants needed for optimal attraction of the mosquitoes. This can be overcome by using larger sachets. It is also possible that the carboxylic acids present in the blend disperse in smaller quantities through LDPE than from the nylon strips. The odor baits used for tsetse flies consist of entirely different chemicals (mostly 1-octen-3-ol, 4-methyl phenol, and 3-n-propyl phenol) (Bursell et al., 1988; Vale, 1993), which may disperse more readily through LDPE than the mosquito kairomones.

From our high throughput work on candidate attractants of *An. gambiae* (Verhulst et al., 2011a; Smallegange et al., 2012), we selected isovaleric acid, 4,5-dimethylthiazole, 2-methyl-1-butanol, and 3-methyl-1-butanol because of the increased attractiveness expressed when these compounds were added to the standard blend. It was, therefore, surprising that in contrast to previous results, here under semi-field conditions both isovaleric acid and 4,5-dimethylthiazole caused an inhibitory effect. Even more so, when two or more of these compounds were added to the standard blend, the effect of each compound individually was cancelled out, or the entire blend seemed repellent, causing most mosquitoes to avoid entering any of the traps. With all four compounds added to the standard blend, the overall response of the mosquitoes was significantly reduced. It is possible that when employing higher dilutions of each of these compounds when studying the effect of multiple compounds, the kairomonal potency of the blend might be further enhanced. We used an industrially-produced batch of 2-methyl-1-butanol. This compound is known to exist of two isomers (R and S). In our study, we used a racemic mixture. It is possible that only one of the isomers would have evoked a behavioral effect in the mosquito, which should be followed up in a future study. The results from the dose-effect study with 3-methyl-1-butanol show that highly diluted concentrations cause attraction of *An. gambiae*, while higher doses cause inhibition. This result is important for the development of attractive odorant blends for mosquitoes. For example, the mosquito repellent DEET is repellent over a wide range of concentrations, but becomes attractive at very low concentrations (Mehr et al., 1990), and this principle of concentration-dependent dual action might apply to many other odorant cues that affect insect behavior (Smallegange and Takken, 2010).

We examined the response of mosquitoes to candidate odorant blends in traps placed outdoors, next to a house, and traps placed indoors. A recent study showed that catches from indoor and outdoor MM-X traps were comparable (Jawara et al., 2009), suggesting that the mosquitoes perceived the odorant cues from a distance, and would either be caught while approaching the house or after house entry. Although Lwanda and Kigoche are approximately 120 km apart, with different ecologies, the mean number of *An. gambiae* s.l., *An. funestus*, and *Culex* spp. was not much different. Odor-baited traps caught significantly more mosquitoes than unbaited traps, thus demonstrating the attractive effect of the blends under investigation. On average, the trap placed next to a human-occupied bed net caught similar numbers of mosquitoes as the trap next to the dispenser baited with the Mbita blend. Unlike in the Tanzania study, where IB1 was 2–3 times as attractive as the human-odor baited trap (Okumu et al., 2010b), here, IB1 was less attractive than the human-baited trap. This difference between these studies may have been caused by different attractiveness of the human volunteers, environmental differences, or both. Each is likely to affect trap catches. The differences were small, though, and the overall result shows that the three synthetic blends approached the attractiveness of the human host (Table 5), with MB being the most attractive blend.

That the majority of the malaria vectors *An. gambiae* s.l. (92.8%) and *An. funestus* (96.8%) collected from Kigoche village was unfed implies that the blends, more so MB that attracted the highest numbers of mosquitoes, mainly target host-seeking mosquitoes. It is not surprising, therefore, that the numbers of mosquitoes attracted to MB were physiologically not significantly different from those attracted to human subjects. However, although MB collected significantly higher fractions of blood fed *An. gambiae* s.l. and *An. funestus* than the trap next to the human-occupied bed net, the catches of mosquitoes displaying this physiological status as well as gravid ones were small.

*Anopheles gambiae s.s.* was the species used in the semi-field study, whereas in the field study *An. arabiensis* dominated the *An. gambiae* s.l. collections. In the past decade, populations of *An. gambiae s.s*. have declined dramatically, presumably as a result of widespread bed net use, whereas populations of *An. arabiensis* have remained similar (Meyrowitsch et al., 2011). The latter species is less affected by the bed nets as it is more exophilic, and less anthropophilic. Nevertheless, *An. arabiensis* is attracted strongly to the synthetic blends, as shown by the Okumu et al. (2010b) study as well as by the current study (Tables 2 and 5). Because *An. arabiensis* can be an important malaria vector and is regularly found in human landing catches (Port et al., 1980), the synthetic blends used here can provide a tool for the sampling of this species. Along the same line, the important malaria vector *An. funestus* was also caught in both villages, responding significantly more strongly to the Mbita blend than to the other blends or the human host. As other, non-anopheline mosquitoes were also collected, the synthetic blends, notably MB, should be considered as attractants for a wide range of mosquito species, including other disease vectors.

We conclude that the multiple-component odorant blends described in this study can be considered to replace traditional malaria vector sampling tools such as CDC light traps and the human landing catch. Next to the ease of use, the availability of a “standard” host, used in multiple locations simultaneously over a study area, is a distinct advantage in providing unbiased data on relative mosquito densities. This will be of advantage for malaria epidemiological studies, but also cost effective. Because the baits are also effective outdoors, they open up a novel avenue for sampling of outdoor biting malaria vectors, which have recently been reported to be a major source of malaria transmission (Reddy et al., 2011; Russell et al., 2011).
